# Rapid Turnover of 2-LTR HIV-1 DNA during Early Stage of Highly Active Antiretroviral Therapy

**DOI:** 10.1371/journal.pone.0021081

**Published:** 2011-06-08

**Authors:** Weijun Zhu, Yanmei Jiao, Rongyue Lei, Wei Hua, Rui Wang, Yunxia Ji, Zhiying Liu, Feili Wei, Tong Zhang, Xuanlin Shi, Hao Wu, Linqi Zhang

**Affiliations:** 1 AIDS Research Center, Institute of Pathogen Biology, Chinese Academy of Medical Sciences and Peking Union Medical College, Beijing, China; 2 Center for Infectious Diseases, Beijing You-An Hospital, Capital Medical University, Beijing, China; 3 Comprehensive AIDS Research Center and Research Center for Public Health, School of Medicine, Tsinghua University, Beijing, China; New York Blood Center, United States of America

## Abstract

**Background:**

Despite prolonged treatment with highly active antiretroviral therapy (HAART), the infectious HIV-1 continues to replicate and resides latently in the resting memory CD4+ T lymphocytes, which blocks the eradication of HIV-1. The viral persistence of HIV-1 is mainly caused by its proviral DNA being either linear nonintegrated, circular nonintegrated, or integrated. Previous reports have largely focused on the dynamics of HIV-1 DNA from the samples collected with relatively long time intervals during the process of disease and HAART treatment, which may have missed the intricate changes during the intervals in early treatment.

**Methodology/Principal Findings:**

In this study, we investigated the dynamics of HIV-1 DNA in patients during the early phase of HARRT treatment. Using optimized real time PCR, we observed significant changes in 2-LTR during the first 12-week of treatment, while total and integrated HIV-1 DNA remained stable. The doubling time and half-life of 2-LTR were not correlated with the baseline and the rate of changes in plasma viral load and various CD4+ T-cell populations. Longitudinal analyses on 2-LTR sequences and plasma lipopolysaccharide (LPS) levels did not reveal any significant changes in the same treatment period.

**Conclusions/Significance:**

Our study revealed the rapid changes in 2-LTR concentration in a relatively large number of patients during the early HAART treatment. The rapid changes indicate the rapid infusion and clearance of cells bearing 2-LTR in the peripheral blood. Those changes are not expected to be caused by the blocking of viral integration, as our study did not include the integrase inhibitor raltegravir. Our study helps better understand the dynamics of HIV-DNA and its potential role as a biomarker for the diseases and for the treatment efficacy of HAART.

## Introduction

Highly active antiretroviral therapy (HAART) can effectively reduce the human immunodeficiency virus (HIV)-1 to undetectable levels (<50 copies/ml) in the plasma. Early theoretical studies have suggested that both the virions and the productively infected cells have very short lives and hence can be completely eliminated in 2–3 years with HAART [1]–[4]. However, some later reports revealed the presence of provirus that is integrated quiescently within the resting memory CD4 T cells [5]–[9] as well as the persistence of provirus even with prolonged treatment [6], [7], [10]–[12]. Moreover, low levels of continued and residual viral replication have also been reported, despite the complete suppression of plasma viremia with HAART [11]–[16]. It is therefore not surprising that HIV-1 could promptly rebound after the cessation of antiretroviral therapy [17]–[22].

The viral persistence of HIV-1 is usually carried out by its DNA. The HIV-1 DNA has three major forms that reflect the different stages and fates of development during viral replication: 1) the linear nonintegrated form, 2) the circular nonintegrated form, and 3) the integrated provirus. The circular nonintegrated form can be further classified as 1-LTR and 2-LTR based on the number of LTR in HIV-1 DNA. No clear conclusion has been reached for the half-life of the nonintegrated form, as it might be either very short [14], [23]–[25] or very long [11], [26]–[29]. In chronic patients without HARRT, 2-LTR was reported to be stable while 2-LTR was lower in those responded well to treatment [28]. The integrated form, however, has a clearly long half-life [11], [24], [30] and is thus the fundamental constituent of latent reservoir [5], [6], [24], [31], [32].

All the three forms of HIV-1 DNA can be measured by the standard real-time PCR (RT-PCR) or Southern hybridization, which has helped us gain substantial insights into the dynamics and the relative contributions of these forms to HIV-1 replication and latency during the disease progression and the HAART treatment. It has been proposed to use one or the combination of these forms of HIV-1 DNA as biomarkers to monitor the viral replication as well as to evaluate the efficacy of various antiretroviral regimens in infected individuals. However, most previous reports have been focusing on the dynamics of HIV-1 DNA with samples collected with relative long time intervals during the disease progression and treatments [28], [30], [33]–[36]. These studies may have missed the intricate changes in the dynamics of HIV-1 DNA during the early treatment, although they have significantly advanced our understanding of the dynamics over a relatively long time period [24], [35], [37]. Furthermore, better quantification and controls are needed to strength the conclusions from these reports.

In this study, we investigated the dynamics of integrated, 2-LTR circular, and total HIV-1 DNA in patients during early phase of HARRT treatment. Using optimized RT-PCR to measure both the target HIV-1 DNA and the input number of cells, we observed significant changes in 2-LTR but not in other forms of HIV-1 DNA during the early stage of treatment. We further measured the doubling time and the half-life of 2-LTR, whose potential correlations with the baseline and the rate of changes in plasma viral load and various CD4+ T-cell populations were also evaluated. Sequencing analysis of 2-LTR was conducted for selected samples to monitor their longitudinal changes over time. Our findings help us clearly understand the replication and latency of HIV-1 during early stages of treatments.

## Results

### Rapid changes in 2-LTR concentration during early treatment

To quantify the HIV-1 DNA and the cellular CCR5 targets in the patients' peripheral blood mononuclear cells (PBMCs) using RT-PCR, we applied an accurate, stable and cost-effective standard based on bacteriophage M13, as being described previously by us [38]. This M13 phage-based standard can be performed without DNA extraction, as the single-stranded circular form of DNA is automatically released into the RT-PCR mixture once it is heated to 95°C prior to PCR amplification [38]. In this study, we generated specific M13 phage standards for the total, 2-LTR, and integrated forms of HIV-1 DNA as well as the cellular gene CCR5, as described previously [39]–[42]. These standards were as sensitive, accurate and stable as our previous reports [38].

**Figure 1:**
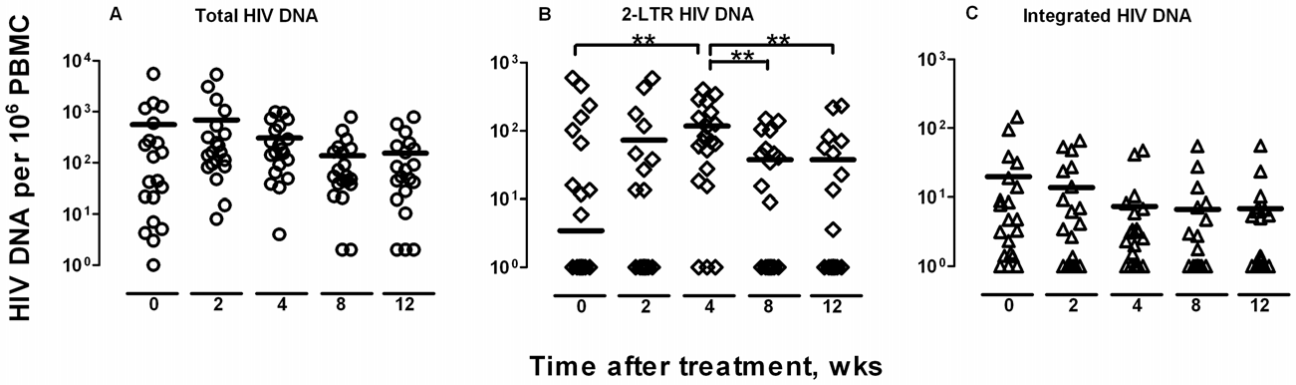
Sequential changes in various HIV-1 DNA in all patients. The total (A), 2-LTR (B) and integrated (C) HIV-1 DNA in 20 patients at week 0, 2, 4, 8, to 12 after treatment were analyzed. The horizontal lines for each time point indicate the median values. Significant differences in median values between different time points were highlighted by asterisk when *p*-value was less than 0.01.

Following these standards, we measured the total, 2-LTR, and integrated HIV-1 DNA in PBMC collected from 20 patients at week 0, 2, 4, 8, and 12 after ARV treatment by RT-PCR. As shown in Figure 1, the 2-LTR underwent rapid changes during the first 12 weeks of treatment. In detail, a rapid rise in 2-LTR was observed up to week 4 of treatment followed by a significant decline during the following weeks. The peak concentration was reached 4 weeks after treatment (Figure 1 B). In contrast, the total and integrated forms of HIV-1 DNA remained stable (Figure 1 A, C). At the same time, all patients demonstrated rapid declines in HIV-1 RNA levels, which even became undetectable in some patients (<40 copies/ml). The integrase inhibitor raltegravir was not included in this study (Table 1).

**Table 1:** The patients' HIV-1 RNA loads, CD4 cell counts, and ART.

			HIV-1 RNA loads (copies/ml)^a^			CD4 cell count (cells/µl)						
Patient	Age	Gender	0 W	4 W	12 W	0 W	2 W	4 W	8 W	12 W	HARRT	Duration of Infection
1	29	M	40,327	1,053	163	288	288	1,126	317	285	AZT+3TC+NVP	na
2	28	M	18,662	176	<40	238	238	334	331	312	D4T+3TC+NVP	3 y
3	37	M	90,589	920	<40	90	90	55	107	108	D4T+3TC+NVP	11 y
4	63	M	27,846	661	74	89	192	139	176	190	D4T+3TC+NVP	na
5	23	M	22,291	902	106	278	278	235	366	357	AZT+3TC+NVP	1 y
6	37	M	462,400	1,768	117	154	154	102	257	289	D4T+3TC+NVP	9 y
7	45	M	56,488	960	258	320	320	411	452	370	AZT+3TC+NVP	5 y
8	39	M	17,838	387	49	237	237	300	282	307	AZT+3TC+NVP	1 y
9	29	M	9,264	<40	57	335	293	225	198	215	AZT+3TC+NVP	2 y
10	42	M	315,132	4,371	304	297	214	170	321	356	D4T+3TC+NVP	2 y
11	35	F	5,144	305	<40	102	116	240	255	206	D4T+3TC+NVP	6 y
12	36	M	1,157,417	6,275	258	329	300	256	265	320	D4T+3TC+NVP	2 y
13	26	M	28,118	335	59	172	203	212	282	184	D4T+3TC+NVP	5 y
14	29	M	33,721	618	<40	229	229	246	248	413	D4T+3TC+NVP	3 y
15	25	M	22,968	142	<40	266	155	276	319	369	D4T+3TC+NVP	na
16	38	M	71,559	681	64	256	340	477	303	320	D4T+3TC+NVP	12 y
17	23	M	1,294	<40	<40	293	293	483	451	536	D4T+3TC+NVP	2 y
18	58	M	14,742	645	<40	239	251	505	317	363	D4T+3TC+NVP	5 y
19	64	M	151,973	4,679	550	218	218	587	447	469	D4T+3TC+NVP	0.5 y
20	34	M	47,952	4,881	199	349	589	605	659	715	D4T+3TC+NVP	na
Median Value	36	-	30,920	792	106	248	238	266	310	320	-	3 y

a Viral load was measured by the Amplicor HIV-1 monitor ultrasensitive Method (Roche), with a detection limit of 40 copies/ml of plasma.

### Dynamics of the 2-LTR, total and integrated HIV-1 DNA during early treatment

**Figure 2:**
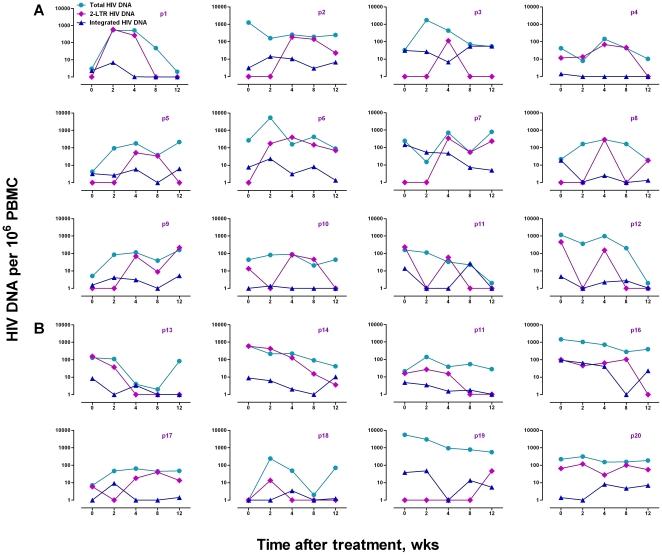
Individual sequential changes in various HIV-1 DNA. The total, 2-LTR and integrated HIV-1 DNA in each of the 20 patients during the first 12 weeks of treatment were quantified by real-time PCR. The patients are divided into two groups, Group A with significant increases in 2-LTR and Group B without.

We analyzed the dynamic changes in 2-LTR in each patient using a simple mathematical model [2], [28], [37]. We found that the rapid changes in 2-LTR was largely contributed by 12 (Group A) out of the total 20 patients, while the other 8 patients (Group B) had relatively stable or gradually reduced 2-LTR (Figure 2). The average doubling time was 0.480 week or 3.360 days (Table 2), which is about 10% of that of the total CD4+ T cells in HIV-1 infected patients but only about 2% of that in healthy individuals [43]. It suggests that rapid increase in 2-LTR on treatment is not associated with the rapid proliferation of cells containing 2-LTR that should only decrease the concentration as cells divide. Rather, the rapid increases may be caused by the quick redistribution of 2-LTR-containing cells during early treatment. Furthermore, the half-life of 2-LTR was estimated, based on the rates of decreases, to be an average of 1.174 week or 8.218 days (Table 2), which is similar to that in a recent report with both acutely and chronically infected patients [44] but is clearly longer than those in the reports with the *in vitro* cultured T cell lines [14], [45]. Nevertheless, the rapid changes in 2-LTR during the early treatment did suggest the continued existence of 2-LTR-bearing cells in peripheral blood. It is currently unknown whether these cells were newly infected during the treatment or they were just redistributed from the lymphoid organs where they had been infected and trapped before the treatment. As our study did not include the integrase inhibitor raltegravir, the rapid changes in 2-LTR are not expected to be caused by the blocked integration [44], [46].

**Table 2:** Estimated doubling time or half-life of 2-LTR HIV DNA (weeks).

Patient	Doubling time	Half life^a^
1	0.217	(0.624)
2	0.265	(2.634)
3	0.293	(0.586)
4	1.571	(1.312)
5	0.351	(1.406)
6	0.462	(3.177)
7	0.237	(1.494)
8	0.245	(0.490)
9	0.327	(1.350)
10	0.313	(0.534)
11	0.340	(0.254)
12	0.275	(0.226)
Mean ± Standard deviation	0.480±0.037	(1.174)±0.935

a The slope of the increase rate of was negative, and the number inside the parentheses indicates the half-life.

**Figure 3:**
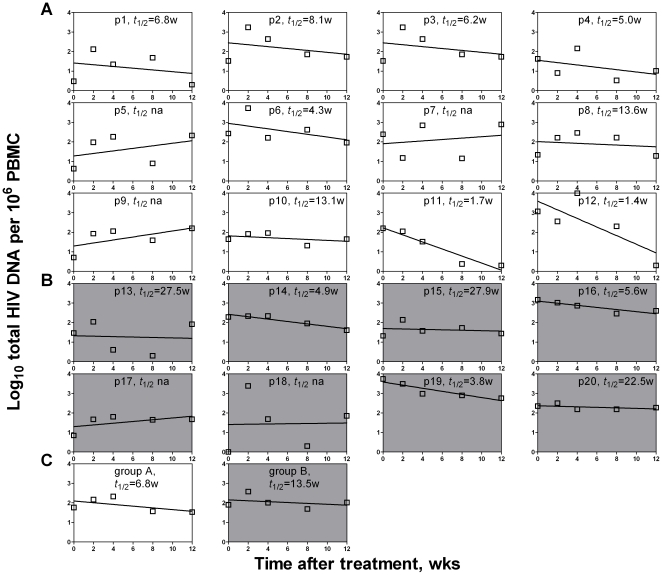
Linear regression analysis on the decay rate of the total HIV DNA. During the first 12 weeks of treatment, each patient in Group A (A) and Group B (B) was analyzed, as well as the averages for the two groups (C). Three patients (p5, p7 and p9) in Group A and two (p17 and p18) in Group B did not have applicable decay rates (*t*_1/2_ *na*) and were therefore not included in the calculation of average shown in panel C.

**Figure 4:**
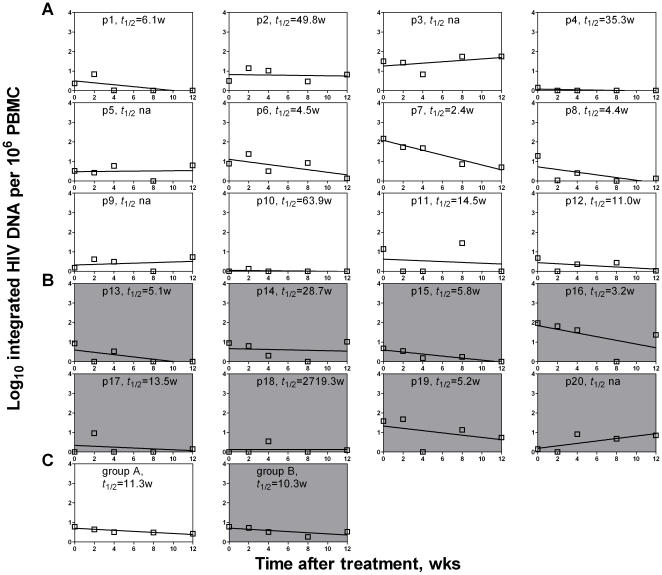
Linear regression analysis on the decay rate of the integrated HIV DNA. During the first 12 weeks of treatment, each patient in Group A (A) and Group B (B) was analyzed, as well as the averages for the two groups (C). Three patients (p3, p5 and p9) in Group A and one (p20) in Group B did not have applicable decay rates (*t*_1/2_ *na*) and were therefore not included in the calculation of average shown in panel C.

We then analyzed the dynamics of total and integrated forms of HIV-1 DNA for each patient. The majority of patients in both Group A and B showed significant declines in total and integrated HIV-1 DNA during the first 12 weeks of HAART (Figures 3 and 4). The average half-life of total HIV DNA for Group A patients is about 3.9 weeks, compared with Group B's about 7.9 weeks (Figure 3). Similar to the total HIV DNA, the integrated HIV DNA's decay half-life was an average of 7.2 weeks for the patients in Group A and 7.1 weeks for the patients in Group B (Figure 4). These results are similar to those of studies with early treatment [3], [37], [47], but significantly shorter than those of studies with extended periods of treatment [12], [27]–[29]. Our results suggest that the total form and the integrated form of HIV-1 DNA decay with similar kinetics during early treatment, and that some of the HIV-1 DNA becomes persistent during late treatment without significant decay within the latently infected cells [6], [7], [10]–[12], [24].

### The rapid changes in 2-LTR are not associated with the baseline and the rate of changes in plasma viral load and various CD4+ T-cell populations

**Figure 5:**
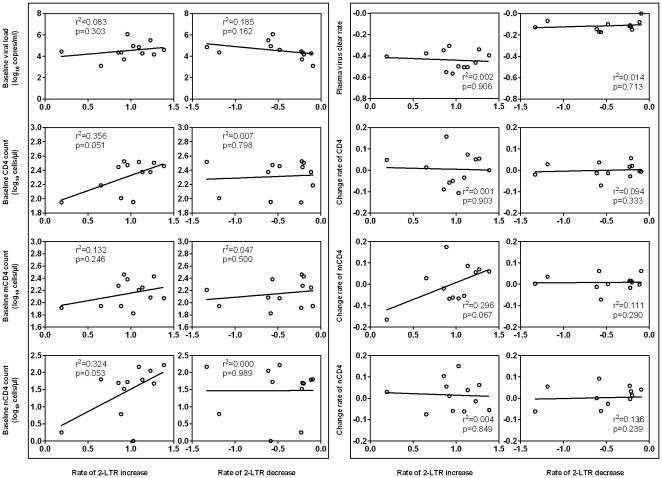
Correlation analysis for the change rates of 2-LTR. Relationships between baseline parameters and the later 2-LTR change rates were shown in the left panel, and the correlations between later change rates were shown in the right panel. Inside each panel, the correlations between the rate of changes in 2-LTR and the baseline or the change rate in plasma viral load, the total CD4^+^, memory (RO^+^) (mCD4) and naïve (RA^+^) CD4^+^ (nCD4) T cell count for the 12 patients in Group A were shown. Rate of 2-LTR increase or decrease was indicated as below.

To study the potential biological implications of rapid changes in 2-LTR during early treatment, we conducted correlation analyses with baseline characteristics and the rates of changes in plasma viral load, total CD4, memory CD4 (mCD4) and naïve CD4 (nCD4) T-cell populations for all patients in Group A, using Spearman's correlation coefficient. As shown in the left panel of Figure 5, no significant association was identified between the rate of changes in 2-LTR and the baseline plasma viral load and various CD4+ T-cell populations. Furthermore, the rate of changes in 2-LTR was not significantly associated with the rate of changes in plasma viral load and various CD4+ T-cell populations (Figure 5, right panel). These results indicate that the rapid changes in 2-LTR are not dependent on the suppression of plasma viral RNA or the increases in peripheral CD4 T-cells during the first 12 weeks of treatment.

### Rapid changes in 2-LTR concentration did not affect the junction and flanking regions of 2-LTR

**Figure 6:**
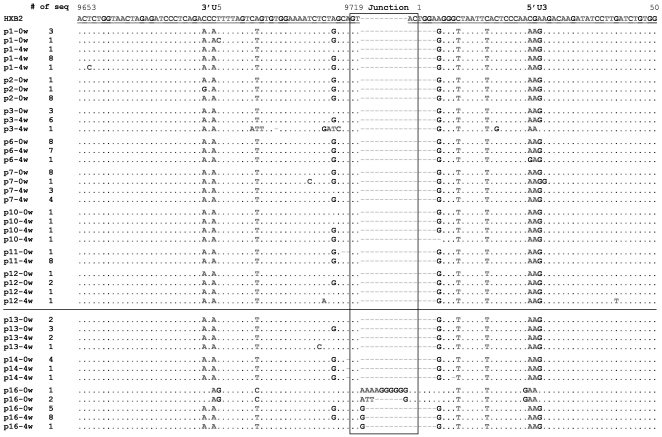
Sequence analysis of 2-LTR circle junction from selected patients in Group A and B. The circle junction, 3′U5 and 5′U3 were aligned and numbered against HXB2. PCR products amplified from samples at week 0 and 4 were cloned and sequenced. Sequences for patients from group A are above the line while those from group B are under the line.

To identify the source of rapidly increased 2-LTR in peripheral blood, we amplified the junction region of 2-LTR directly from the PBMC and analyzed its sequences in the samples of when 2-LTR was at the baseline and when 2-LTR was at peak after HAART treatment. No significant differences were observed for the junction and flanking regions of 2-LTR between the two time points for all patients, except for the patient 16 in Group B (Figure 6). This patient had varied lengths of 2-LTR sequences with a dominant form of one single Guanine nucleotides (G) at the junction (5/8) when 2-LTR was at the baseline level. Four weeks later, this dominant sequence had completely replaced others (9/9) (Figure 6). These results suggest that the rapid changes in 2-LTR concentration in peripheral blood are not correlated with the changes in 2-LTR sequence during early treatment, which makes it extremely hard to predict the source of the 2-LTR-bearing cells in the peripheral blood during early HAART treatment.

### Rapid changes in 2-LTR concentration are not associated with the plasma lipopolysaccharide (LPS) levels in early HAART treatment

**Figure 7:**
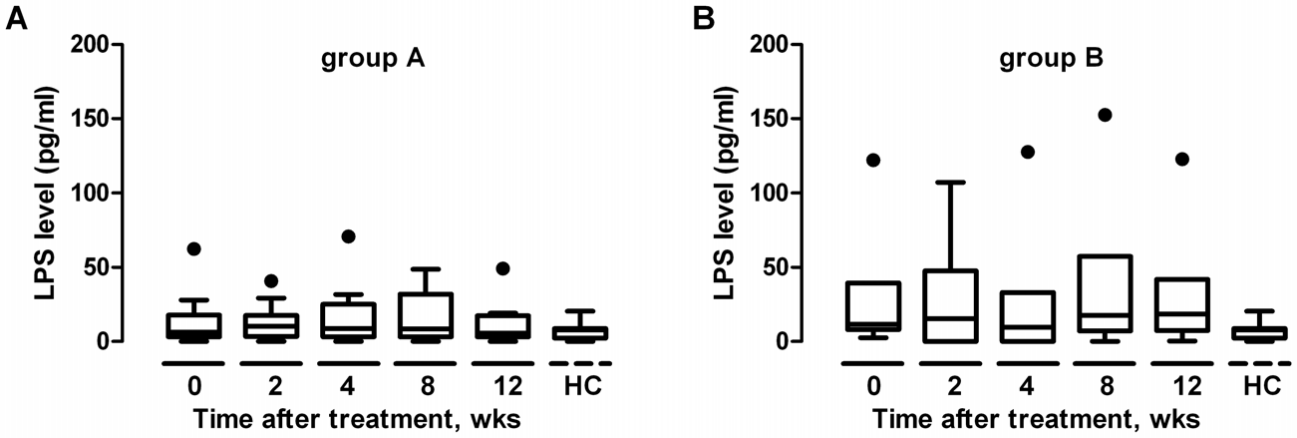
Changes in plasma LPS. Plasma LPS concentrations were determined during the first 12 weeks of treatment for Groups A and B, compared with those from healthy controls. Box plots indicate the median, the 25th and the 75th percentiles concentrations for the samples at week 0, 2, 4, 8 and 12. Outliers with exceptionally high concentration are indicated by dots. HC, health control, n = 10.

It has been reported that patients with chronic HIV infection had significantly higher levels of plasma LPS than the acutely infected patients and normal individuals, which led to generalized immune activation [48], [49] and likely the trapping of many 2-LTR-containing cells in the lymphoid tissues. Based on this notion, we sought to measure the levels of plasma LPS during the first 12 weeks of HAART treatment. As shown in Figure 7, no significant changes in plasma LPS levels were observed for both Group A and Group B, nor were any significant differences in plasma LPS levels between these two groups. LPS levels in these two groups were higher than those in health controls, however, the differences were not statistically significant. It suggests that 12 weeks of HAART treatment does not affect the LPS levels and the rapid changes in 2-LTR during this treatment period is caused by other factors.

## Discussion

In this study, we investigated the dynamics of 2-LTR, integrated and total HIV-1 DNA in patients during the first 12-week HAART treatment using an optimized RT-PCR method with novel M13 phage-based standards. Significant changes in 2-LTR concentration were observed in 60% of the patients. Kinetic analysis revealed that the doubling time and the half-life of 2-LTR were significantly different than those of CD4+ T cells [43], [50]–[52] but not correlated with the baseline and rate of changes in plasma viral load and various CD4+ T-cell populations. Future studies would be needed to evaluate the changes of 2-LTR concentration in various T cell populations and its relationship with dynamics of these cells. Lastly, no significant changes in 2-LTR sequences or plasma LPS levels were observed during the same treatment period.

To our knowledge, this study is the first to demonstrate that 2-LTR undergoes rapid changes in the peripheral blood during early treatment of HAART. In pursuing the source of 2-LTR-bearing cells in the peripheral blood, we first excluded the involvement of 2-LTR accumulation in the infected cells since the integrase inhibitor raltegravir was not included in this study. The elevated levels of 2-LTR might be caused by the continued HIV-1 infection of target cells in the peripheral blood despite of substantial drop in plasma viral RNA. However, this hypothesis can not explain the subsequent decline in 2-LTR, as continued infection would lead to persistent burst of 2-LTR. It is also possible that the 2-LTR-bearing cells were part of the cells that had been trapped in the lymphoid tissues by the generalized immune activation with HIV-1 infection. This re-distribution hypothesis is consistent with the observation of rapid and transient increases in memory CD4+ T-cells during early HAART treatment [50]–[52]. However, future investigation is required to figure out the exact source of rapid changes in 2-LTR in the peripheral after early treatment.

In summary, our study revealed the rapid changes in 2-LTR concentration in a relatively large number of patients during early HAART treatment, while the total and integrated forms of HIV-1 DNA remained stable. These rapid changes might be associated with the rapid redistribution of memory CD4+ T cells that had been previously trapped in various lymphoid tissues. If this hypothesis were to be confirmed by future studies, the 2-LTR concentration in peripheral blood could serve as an important biomarker for HIV-1 infection and treatments in addition to the plasma viral load and CD4 count since 2-LTR has already been used to indicate the actively viral replication or the potency of antiretroviral drug such as the integrase inhibitor raltegravir [44], [46]. Furthermore, our longitudinal analysis of 2-LTR, integrated and total forms of HIV-1 DNA also provided substantial insights into the dynamics of HIV-1 over the course of infection, which may help us better understand the pathogenesis of the diseases and develop more effective treatment strategies.

## Materials and Methods

### Ethics statement and study subjects

This study was approved by the Ethical Committee at Beijing You'an Hospital and written informed consent was obtained from all participants. A total of 20 HIV-1 positive individuals who had not received any antiretroviral therapy prior to this study were enrolled. The demographic, virologic and immunologic characteristics of these subjects are listed in Table 1. All individuals except one were male with ages from 23 to 64. At baseline, they had a median CD4 lymphocyte count of 248 cells/µl and a median plasma viral load of 30,920 copies/ml. All individuals were treated with antiretroviral regimens containing two nucleoside reverse-transcriptase inhibitors plus a non-nucleoside reverse-transcriptase inhibitor, nevirapine. No integrase inhibitors such as raltegravir were used. Blood samples were collected at baseline, at week 2, 4, 8 and 12 after treatment. Twelve weeks after the treatment, all individuals had rapid declines in viral load and a majority of them showed undetectable levels of HIV-1 RNA (<40 copies/ml) in the plasma. Concomitant rise in CD4 T cell count was observed in most of the subjects (Table 1).

### Primers, probes and M13 bacteriophage-containing target sequences as standards for RT-PCR

The primer and probe sequences for RT-PCR assays were optimized based on previous reports [39], [40] and the published sequences of geographical variants in the Los Alamos HIV databases (http://www.hiv.lanl.gov/). The primer and probe sequences as well as their targets of detection are listed in Table 3. As the standard for RT-PCR, we used M13 bacteriophage as a vector to express appropriate HIV-1 DNA and cellular sequences. We chose the chemokines receptor CCR5 as a surrogate to estimate the input number of cells, because each cell contains only one single copy of CCR5 [13], [53]. The absolute number of target sequences could be easily calculated based on the number of plaque forming unit (PFU) of M13 phage, because one phage contains one copy of the genome [38]. To generate standard for the various forms of HIV-1 DNA and the cellular CCR5, the corresponding gene fragments were inserted into the M13 genome. The recombinant M13 phages were propagated in *Escherichia coli* JM109 at 37°C overnight and harvested by centrifugation. The titer of recombinant phage in the supernatant was estimated by serial dilution, followed by the counting of PFU [38].

**Table 3:** Primer and probe sequences.

Primer or probe	Sequence (5′-3′)	Target
MH531	TGTGTGCCCGTCTGTTGTGT	Total HIV DNA^a^
MH532	GAGTCCTGCGTCGAGAGAGC	
LRT-P*	FAM-CAGTGGCGCCCGAACAGGGA-TAMRA	
MH535	AACTAGGGAACCCACTGCTTAAG	2LTR circle^a^
MH536	TCCACAGATCAAGGATATCTTGTC	
MH603r^d^^,^ *	FAM-AAAGCTTGCCTTGAGTGCTTCAAGTAGTGT-TAMRA	
L-M667	ATGCCACGTAAGCGAAACTGGCTAACTAGGGAACCCACTG	Integrated HIV DNA^b^
Alu1	TCCCAGCTACTGGGGAGGCTGAGG	(first-round PCR)
Alu2	GCCTCCCAAAGTGCTGGGATTACAG	
Lambda T	ATGCCACGTAAGCGAAACT	Integrated HIV DNA
AA55M	GCTAGAGATTTTCCACACTGACTAA	(second-round PCR)
LRT-P*	FAM-CAGTGGCGCCCGAACAGGGA-TAMRA	
CCR5-f	CAAAAAGAAGGTCTTCATTACACC	CCR5^c^
CCR5-r	CCTGTGCCTCTTCTTCTCATTTCG	
CCR5-p*	FAM-GCGAGTCCTGCCGCTGCTTGTCATGGTCCTCGC-DABCYL	

*: probe sequence;

a : Butler et al., 2001;

b : Brussel and Sonigo, 2003;

c : Huang et al., 1996; Lai et al., 2003;

d : reverse complement as in the reference.

### Quantification of HIV-1 DNA and cellular CCR5 with RT-PCR

The total DNA from patients' PBMCs was extracted using QIAamp DNA blood mini kit (Qiagen), eluted in DNase-free water, and stored at −80°C until use. RT-PCR was performed in 25-µl solution containing 2.5 µl of DNA target, 12.5 µl of Gene Expression Master Mix (Applied Biosystems 4369016), 1 µM of primers and 0.2 µM of probe under the following conditions: 95°C for 10 min, followed by 40 cycles of 95°C for 15 s and 60°C for 1 min in ABI 7500 PCR machine (Applied Biosystems). For the integrated HIV-1 DNA, the first round of PCR was conducted with the following conditions: 95°C for 8 min, and then 12 cycles of amplification at 95°C for 10 s, 60°C for 10 s, and 72°C for 170 s. One-tenth of product from the first round PCR was transferred to a new tube for the following RT-PCR quantification analysis. The PCR signal from the first round without the Alu primers was subtracted from the total signals prior to the estimate of copy numbers. For the total and 2-LTR HIV-1 DNA as well as the CCR5 gene, only one round of RT-PCR was performed. A standard curve was created for each run in a 7-log-unit range by 1∶10 serial dilutions.

### Immunologic analysis of T cell populations in the blood

The CD3, CD4, CD45RA and CD45RO subpopulations in peripheral blood samples from HIV-1 infected individuals were quantified with four-color flow cytometry using the following monoclonal antibodies (mAbs): CD3-PerCP, CD4-FITC (BD Bioscience, CA, USA), CD45RA-APC and CD45RO-PE (eBioscience, CA, USA). Naive cells were defined as CD4+CD45RA+, whereas memory cells were defined as CD4+CD45RO+. The analyses were performed using FACSCalibur and CELLQuest software (Becton Dickinson, San Jose, CA).

### Sequencing analysis of 2-LTR PCR products

The extracted DNA samples from PBMC at week 0 and 4 were amplified by PCR before being cloned into the pMD18-T vector (Takara, Japan). Ten clones of each sample were sequenced and those with sequences available at two time points were analyzed using the software Clustal X version 1.83 [54].

### Measurement of bacterial lipopolysaccharide (LPS) levels in the plasma

The plasma was heat-inactivated at 70°C for 10 min before being diluted at 1∶5 in endotoxin-free water. The plasma concentration of LPS was measured and calculated using the LPS ELISA Kit (R&D Systems).

### Statistical Analysis

All data were analyzed using the software of SPSS version 16.0. The correlation between two parameters was determined using the Spearman's correlation coefficient. It was considered as statistically significant when *P*<0.05. The Wilcoxon paired test was used to compare the longitudinal changes and the Mann-Whitney *U* test was used to compare the medians between different groups. The doubling time (log_10_2/slope) or half-life (−log_10_2/slope) of HIV-1 DNA was calculated following previously described methods [2], [28], [37].
